# Scale-up considerations for monoclonal antibody production process: an oxygen transfer flux approach

**DOI:** 10.1186/1753-6561-7-S6-P49

**Published:** 2013-12-04

**Authors:** Laura Gimenez, Claire Simonet, Laetitia Malphettes

**Affiliations:** 1 BioTech Sciences, UCB Pharma SA, Braine l'Alleud, Belgium

## Background

When scaling up a monoclonal antibody (mAb) production process in stirred tank bioreactor, oxygen transfer is probably one of the most challenging parameters to consider. Approaches such as keeping constant specific power input or tip speed across the scales are widely described in the literature and are often based on the assumption that mammalian cells are sensitive to shear stress.

However, with the high cell densities reached in modern processes, such scale-up strategies can lead to relatively high gas flow rate to compensate low agitation speed which could be detrimental to cells in its own right.

As an alternative, we explored a scale-up strategy based on the overall oxygen transfer flux (OTF) required by the cell culture process. OTF was defined as directly proportional to oxygen transfer coefficient (k_L_a) and oxygen enrichment in the gas mix. This way the overall gas flow can be kept at relatively low values, while satisfying the oxygen requirements of a high cell density culture.

## Materials and methods

Process scale-up between 3 different stirred tank bioreactors was studied: a 2 L glass bioreactor (Sartorius Stedim Biotech) equipped with one 3-segment blade impeller, a 10 L glass bioreactor (Sartorius Stedim Biotech) equipped with two 3-segment blade impellers and a 80 L stainless steel bioreactor (Zeta Biopharma) equipped with two elephant ear impellers.

Oxygen transfer coefficients (k_L_a) were determined for the chemically defined production medium, using the dynamic technique of oxygen adsorption. The statistical analysis software JMP (SAS) was then used in order to express k_L_a's according to the following equation: k_L_a = A * (P/V) ^α ^* Vs^β^, P/V being volumetric power input [W.m^-3^] and Vs being superficial air velocity [m.s^-1^], and to analyze our results.

Oxygen transfer flux was defined as followed: OTF = k_L_a * (%O_2 _in the gas mix/% O_2 _in air).

For cell culture experiments, bioreactors were inoculated with a CHO cell line producing a mAb. Cells were cultivated in chemically defined media for a 14-day fed-batch process. The culture was controlled to maintain the desired process parameters (temperature, pH, dO_2 _and glucose concentration). dO_2 _level was maintained using a cascade aeration. Viable cell density (VCD) and viability were monitored by Trypan blue dye exclusion using a Vicell XR (Beckman Coulter). Glucose and lactate concentrations were determined using a Nova Bioprofile 400 analyzer (Nova Biomedical). Offline dissolved CO_2 _and osmolality were measured with a Nova Bioprofile pHox (Nova Biomedical) and Osmo 2020 (Advanced Instrument) analyzers respectively. mAb concentrations were determined by Protein A HPLC.

## Results

### k_L_a mapping of 2 L, 10 L and 80 L bioreactors

The 2 L and 10 L bioreactors were characterized for a range of superficial gas velocity going from 5.0 × 10^-5 ^to 4.0 × 10^-4 ^m.s^-1 ^and the 80 L for a range going from 2.0 × 10^-4 ^to 1.2 × 10^-3 ^m.s^-1^. Specific power input was ranged from 10 to 90 W.m^-3 ^for the 2 L bioreactor, 20 to 130 W.m^-3 ^for the 10 L bioreactor and 5 to 80 W.m^-3 ^for the 80 L bioreactor. Models were generated with JMP and gave the following equations for k_L_a [s^-1^]:

2 L bioreactor: k_L_a = 6.37 × 10^-2 ^* (P/V)^0.28 ^* Vs^0.59 ^(R^2 ^= 0.98, Prob>F: <0.0001)

10 L bioreactor: k_L_a = 4.07 × 10^-2 ^* (P/V)^0.55 ^* Vs^0.67 ^(R^2 ^= 0.91, Prob>F: <0.0001)

80 L bioreactor: k_L_a = 5.53 × 10^-2 ^* (P/V)^0.72 ^* Vs^0.77 ^(R^2 ^= 0.92, Prob>F: <0.0001)

### Scale-up of aeration and agitation strategy of a monoclonal antibody production process using a constant OTF approach

The cell culture process was initially developed at 2 L and 10 L scale. Maximum Oxygen Transfer Flux was determined at maximum cell density for these two scales. This maximum OTF was kept constant for scaling up to 80 L (Table 1). From k_L_a mapping of the 80 L bioreactor, appropriate P/V, Vs and O_2_% values were chosen in order to reach the target OTF.

**Table 1:** Determination of aeration and agitation strategy in the 80 L bioreactor, based on the maximum OTF required by the cells at 2 L and 10 L scales.

	2 L	10 L		80 L
**P/V [W.m^-3^]**	30	69		80

**Vs [×10^-4 ^m.s^-1^]**	0.94	3.53		4.03

**k_L_a [×10^-3 ^s^-1^]**	0.70	1.43		3.85

**%O_2 _in gas mix**	74	90		30

**OTF max [×10^-3 ^s^-1^]**	2.44	6.11	Target OTF for 80 L = 10 L OTF →	5.55

**Figure 1:**
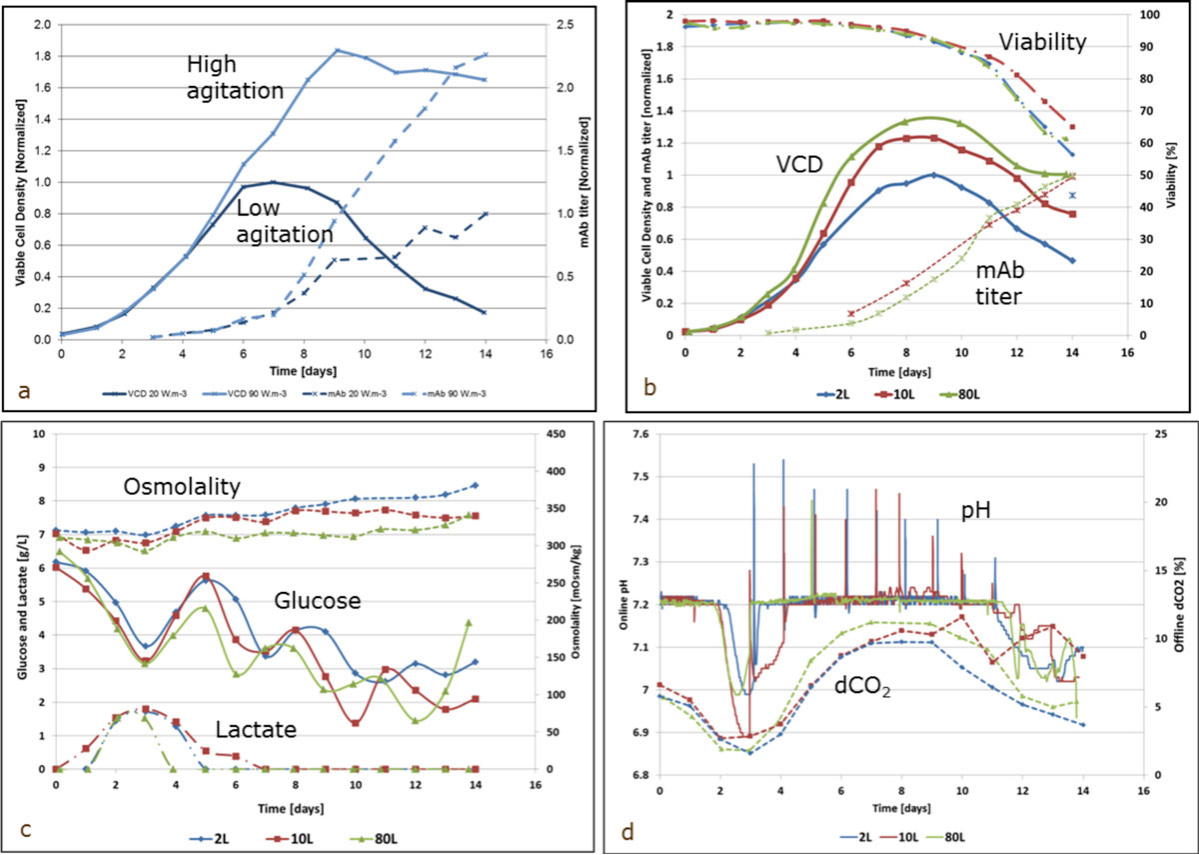
**Cell culture process performance at 2 L, 10 L and 80 L scale**. a) Impact of agitation speed on VCD and mAb titer at 2 L scale. b) Comparison of VCD, viability and mAb titer obtained in 2 L, 10 L and 80 L bioreactors. c) Comparison of osmolality, glucose and lactate profiles obtained in 2 L, 10 L and 80 L bioreactor. d) Online pH and dCO_2 _levels obtained in 2 L, 10 L and 80 L bioreactors.

To confirm that high specific power input are well tolerated by CHO cells, the fed-batch process was first run in two 2 L bioreactors (Figure 1a). Agitation speed was set at 250 rpm (20 W.m^-3^) in the first bioreactor and at 400 rpm (90 W.m^-3^) in the second bioreactor. In the high agitation condition, the maximum VCD was 1.8-fold higher, viability remained above 80% (versus 60% in the low agitation condition) and mAb titer was 2.2-fold higher.

Our model fed-batch process was then run in our 80 L bioreactor, using the aeration strategy defined in Table 1. Figure 1b, c and 1d show that the process was successfully scaled-up from 2 L and 10 L to 80 L bioreactor.

## Conclusions

Thanks to extensive characterization of aeration conditions in 2 L, 10 L and 80 L bioreactors, the oxygen transfer flux approach enabled to have a sufficient aeration and comparable process performance across the scales, including dCO_2 _profile. The same strategy will be used for further scale-up of the process to 2000 L. However, the results also revealed that our 2 L scale model should be re-assessed to become more predictive of 10 L and 80 L scales.

